# Framework for standardizing digital health in resource-constrained settings: a case study of Uganda’s digital health communication infrastructure

**DOI:** 10.1093/oodh/oqae018

**Published:** 2024-06-05

**Authors:** Andrew Alunyu Egwar, Mercy Rebekah Amiyo, Josephine Nabukenya

**Affiliations:** Department of Computer Engineering and Informatics, Busitema University, Tororo, Uganda; Department of Information Systems, Makerere University, Kampala, Uganda; Department of Information Systems, Makerere University, Kampala, Uganda

**Keywords:** communication infrastructure, digital health, eHealth, interoperability, standards, standardization framework, ICT network

## Abstract

Uganda has several digital health interventions, but most are not interoperable, failing the goal of digitizing the healthcare environment. One major reason for this failure is that the implementations are not based on a common set of standards formally agreed upon by the country’s health ministry. Therefore, Uganda and other countries that have fallen behind in standardizing their health systems need to implement and fast-track the formulation of contextual digital health standards. This study followed a design science approach to develop a framework for standardizing the digital health communication infrastructure. The design went through three cycles of design and improvement. Results show that 17 digital health stakeholders in Uganda who evaluated the framework agreed that they are suitable to guide standardization activities in Uganda. This study argues that other countries with digital health challenges similar to those in Uganda can adopt the framework.

## INTRODUCTION

Standardization means the ‘*activity of establishing, with regard to actual or potential problems, provisions for common and repeated use, aimed at the achievement of the optimum degree of order in a given context*’ [1]. The word ‘standardization’ is applied in various domains to mean/explain different things [2]. For example, while to some, the word standardization implies the use of the standard, to others, it means adopting the same procedures or methods to harmonize work processes for compatible outputs [2]. This study adopts a holistic definition of standardization by Costello and Parker [3], who define standardization as the process of development and consistent implementation of standards and other technical documents. However, most standardization discussions have often focused only on the standards development process, and little attention is paid to the standard’s implementation, compliance monitoring and review.

Standardization consists of formulating, issuing and implementing standards [4, 5]. Consistent implementation is achieved through the adoption and use of the standard for its intended purpose. Consistent use of a health standard is determined by ensuring effective use across a health system [6]. This means that complete standardization of the digital health communication infrastructure (DHCI) should extend developing standards (development) to consistent deployment and effective use of the set standards by all health facilities/healthcare organizations participating in the connected health system. However, this layer of standardization only caters to the technical interoperability of digital health (DH) systems, excluding syntactic and semantic components. Whereas a complete standardization of DH (covering technical, terminology and messaging standards) is required to ensure the attainment of an interoperable DH ecosystem [7], DHCI standardization presented in this study contributes only a fraction to the realization of a connected and interoperable health system. Therefore, to completely address the interoperability challenge in a low- middle-income country (LMIC) like Uganda, there is a need for a comprehensive standardization of terminologies, messaging and the technical ICT infrastructure that supports connectedness. Achieving a level of standardization that will deliver interoperability of DH systems ‘*involves both a process for establishing and maintaining the standards and an organizational infrastructure for implementing that process*’ [4]. Regarding DHCI, the framework should address the process of establishing contextual standards and guide implementation, maintenance and the requisite infrastructure/resources.

The standards development activity in the standardization process is protracted, often taking between 18 and 36 months for a standards development organization (SDO) to develop an international standard [7]. This is because standardization is a complex and challenging process [8, 9], requiring many stakeholders to agree by consensus. The European Committee for Standardization (CEN) confessed that the complexity of health informatics (HI) standardization makes their work much harder regarding standardization and communication about standards [10]. Therefore, an LMIC like Uganda or any other country that would like to fast-track standardization activities (standards development, implementation and monitoring) after they have already wasted time deploying several disintegrated DH systems lacking in interoperability would require a systematic procedure to do so. However, such a guide is lacking in aiding resource-constrained settings like Uganda in developing and/or implementing standards for their contextual environments. In fact, one of our earlier studies showed that LMICs lack frameworks, models or structured methods to adapt standards for the DHCI [11]. This was confirmed by a similar study by Alvarez et al. [12], who found no clear methods for contextualizing global health system guidance. Moreover, the methodological gap persists as countries that have advanced in digital standardization, like Estonia or Portugal, Saudi Arabia and the United Arab Emirates (who scored five on digital health maturity phase in the WHO 2023 survey on the state of digital health [13], have not provided LMICs with foundational models/frameworks for contextualizing digital health standards.

**Figure 1:**
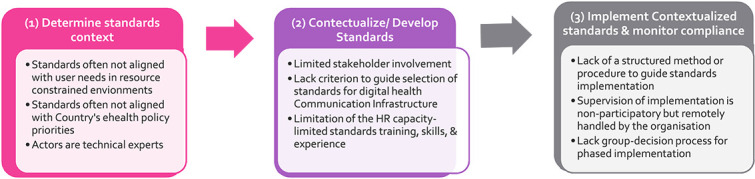
Gaps in the digital health standardization process in LMICs [19]

Although methodologies exist to develop standards for widespread global adoption and use [1, 7, 14, 15], several studies have revealed weaknesses in standardization in LMICs that have hampered their ability to develop/contextualize and implement suitable standards that meet their needs [11, 12, 16–19]. The weaknesses relate to the standard conceptualization, development/determination and standards used. This study conceptualized the challenges as standardization gaps. They were grouped under three main categories (see Fig. 1): (i) gaps that relate to determining the need for a standard (context of the standards), (ii) gaps in the standards adoption/adaption/development process and (iii) weaknesses of the standards implementation process.

Also, literature on standardization shows that the process should attain good standards for development, use and maintenance practices. Although in 2000, the World Trade Organization (WTO) agreed on six principles [20]; CEN argues for eight principles, including voluntary nature, openness and transparency, broad and balanced participation, consensus, consistency, market relevance, benefit for society and wide geographical relevance [21]. All these principles produce quality standards. However, this study adapted the 12 standardization principles that the Australian Digital Health Agency (ADHA) followed. Australia is a high-income country with better resources. Nevertheless, we argue that the same principles can be applied to other health systems. The principles include openness, transparency, representation, impartiality, consensus, market need and net benefit, timeliness, internationality, compliance, coherence, availability and support [22]. If these 12 principles are followed in developing the standards, such standards would be suited to guide the standardization of the DHCI for Uganda’s health system. In addition, agreed-upon standards must be implemented to realize its true potential. However, implementation is dogged by several organizational factors such as limited resources, expertise and organizational structures [19] required to implement and monitor compliance with the standards. The implementation and compliance monitoring infrastructure of DHCI standards should provide the requisite governance structure and resources to support it.

Therefore, the purpose of this paper is to present a framework for the standardization of the DHCI developed to deliver the 12 standardization principles adopted from the ADHA [22] as it guides the country’s health system to (i) contextualize relevant global standards to their resources needs or develop the standards, (ii) plan and implement full rollout the contextualized standards and (iii) monitor compliance with the standards.

## MATERIALS AND METHODS

### Research approach

The design science approach [23] was followed in describing the standardization framework and process. The *relevance cycle* helped identify DHCI standardization problems and determine requirements. An exploratory study was conducted to identify DH standardization gaps in Uganda’s health system. Linking the gaps through literature, foundational theories/frameworks, and best practices from other countries (*rigor cycle*), the researcher derived requirements for the DHCI standardization framework, which Uganda’s DH stakeholders validated. As agreed upon by DH stakeholders in Uganda, the requirements were then used to develop the framework for the standardization of the DHCI. Finally, during the *design cycle,* the researchers used the approved requirements for DHCI framework components before engaging with domain experts in focus group sessions to scrutinize it for completeness and possible errors.

### Framework development process

The design of the first version of the framework was based on the work of [14], which demonstrated that different standardization domains need to adapt the general standardization framework/process to suit their field. Therefore, this study adopted the framework for standardization in telecommunications and information technology as a foundational model [24] and constructed a framework for standardizing the statistical system [14]. Simultaneously, Sherif’s framework addresses six issues of strategy and tactics. The six issues are as follows: why seek a standard? What is to be standardized? When should standardization occur? How does standardization take place? Who conducts/participates in the standardization activity? Where will the standard be used? Braaksma et al. [14] provided constructs for establishing the need for a standard, development, adoption, dissemination, application, and maintenance and review of the standard. However, neither framework considers standardization resources or even monitors and evaluates the standardization process. These two issues were identified in an exploratory study of Uganda’s health system as critical factors of standardization in low-resource settings [19]. The resource component has subcomponents for identifying and allocating resources like financial, human and standards reference documents (reusable assets). The need arises from the background that resources are essential to the standardization process but are often lacking in resource-constrained settings like Uganda, as revealed by the field study.

Therefore, issues identified from the standardization framework by Sherif [24], constructs from Braaksma et al. [14], and emerging issues from an exploratory study of Uganda’s health system were mapped to standardization phases/activities drawn from our published conceptual model for adaptation of eHealth standards by LMICs [11] to produce phases/constructs of the resulting framework for standardizing the DHCI. We included the resources as a vital component required at all phases of the standardization process. Finally, the successful implementation of any information system artifact greatly depends on rigor in monitoring and evaluating (M&E) its development stages and implementation.

Following the researchers’ initial drafting of the framework, a Delphi technique [25] was employed where two workshop sessions (focused groups) were established involving domain experts and DH stakeholders in Uganda to scrutinize and provide input to the framework to determine its completeness. Table 1 shows the distribution of participants in the two workshops and those who evaluated the final version of the framework presented in this study.

**Table 1:** Distribution of participants in framework development workshops and summative evaluation

The objective for each round of evaluation			Participants in each round of the evaluation	Total #
Development workshops	Round 1	Assess the completeness of the framework’s components to improve/refine them.	UNBS	01
			Researchers	02
	Round 2	To assess the usability of all the components of the framework to produce contextual DHCI standards, guide their implementation and continuous M&E	UNBS	01
			NITA-U	01
			MoICT	01
			MoH	01
			UCC	01
Summative evaluation		Summative validation (1) To confirm the completeness and correctness of the constructs/components of this framework and the relationships between them. (2) To assess stakeholders’ views on the applicability of the DHCI framework to guide the development of contextual standards for Uganda, plan implementation and continuous M&E of stakeholder compliance.	DH users at the health facility	03
			District health officer (DHO)	01
			District biostatistician	01
			NITA-U	02
			MoICT	01
			UNBS	01
			MoH	04
			Implementing partners	02
			Researchers	02

The researcher engaged three domain experts in the first round to evaluate the completeness. Feedback from the evaluators was used to improve the framework. The second round of the DHCI framework evaluation focused on the framework’s usability. In this round, the views from five different domain experts were used to inform a further revision of the framework. Finally, round three of the evaluation exercises (summative evaluation) focused on the applicability of the framework to Uganda regarding usefulness, performance and fit to support the standardization process. This round included evaluation metrics of the previous two rounds, including completeness, correctness and usability. The results are presented in this paper.

### Framework validation

To assess the potentiality of the framework to work for LMICs, the developed framework was validated in Uganda’s health system. The validation was informed by various artifact evaluation works [23, 26], with eight quality attributes being considered: functionality, completeness, consistency, accuracy, performance, reliability, usability and fit with the organization. Furthermore, the study adopted the argument of Venable et al. [26], who proposed that static validation ensures the framework/model’s completeness without requiring execution. They emphasize the importance of considering the artifact’s utility and characteristics during evaluation. Asserting that each evaluation is quite specific to the artifact, its purpose(s) and the purpose(s) of the assessment, which in our case was to contextualize standards for supporting Health Information Exchange (HIE) in Uganda’s health system.

The validation process involved several stakeholders drawn from the Uganda’s Ministry of Health (MoH), Uganda National Bureau of Standards (UNBS), National Information and Technology Authority-Uganda, (NITA-U), Ministry of ICT (MoICT), Uganda Communications Commission (UCC), development partners, implementation partners, and HI researchers in Uganda. Before responding to the validation tool, stakeholders were first introduced to the framework and allowed to practice using the standards adaption/contextualization process to select candidate standards that should be contextualized (see Supplementary Material). They used a general standards criterion, DHCI-specific criteria and resource-related criteria to score on four DHCI design and implementation standards and five security and privacy-related standards. The winning standards in each category were selected for adaptation to the contextual needs of Uganda’s healthcare environment. Furthermore, stakeholders in workgroups exercised by contextualizing standards. The tasks involved adding statements of implementation specifications that reflect the local context and setting minimum specifications for its different components.

Purposive and convenience sampling techniques were used to select participants to respond to the validation tool. The aim of the validation was not to generate results that would be used to create generalizations on the entire population [27] but to assess *the completeness, correctness, usability, perceived usefulness and efficacy/satisfaction* of the framework to produce contextualized standards and guide implementation and compliance monitoring in Uganda’s health system. The sampling technique aimed to effectively use available limited resources to identify and select information-rich individuals with experience and knowledge [27–30] in the validation of DHCI component artifacts.

### Data collection

A structured questionnaire was used to collect validation data from the 17 purposively selected participants shown in Table 1. The tools elicited nominal and qualitative responses. As argued by Pries-Heje et al. [31], the questions were informed by selected IS artifact evaluation metrics [32]. The target population was domain experts and users in Uganda’s digital health space. Statistical and qualitative content analysis methods were followed to analyze the quantitative and qualitative data collected, producing the results presented in the next section.

### Ethical clearance

The Institutional Review Board of the School of Public Health, Makerere University approved the study. In addition, all study participants provided informed consent.

## THE FRAMEWORK FOR STANDARDIZATION OF THE DHCI

### Description of the developed framework

**Figure 2:**
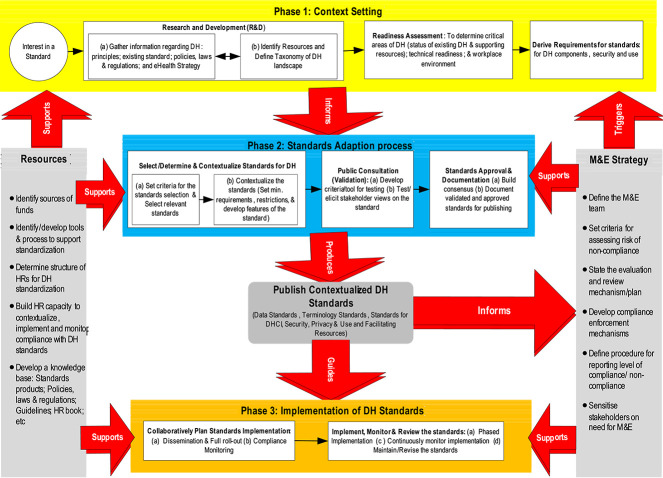
Framework for standardization of the DHCI

The developed framework aimed at fast-tracking standardization/contextualization of the global/international standards for DHCI is shown in Fig. 2.

The framework delineates three major phases of standard contextualization, i.e. standards context setting phase, the standards adaptation process and the standards implementation phase. In addition, the framework provides guidance on monitoring and evaluation and resources to support the standardization process.

#### Phase 1 of context setting

The standardization process begins with an interest in a DH standard from various stakeholders, including users, implementers and policymakers. A standard development workgroup gathers information, defines the standard’s scope and identifies resources needed for its development. The initial stages involve a readiness assessment to determine critical infrastructure areas for funding and standardization. This assessment includes determining the status of existing eHealth CI, technical readiness and workplace environment readiness. Phase 1 concludes with the derivation of requirements for the standards to be adopted or adapted for Uganda, focusing on standards for all components of the DHCI.

#### Phase 2 of standards contextualization

The standards development workgroup identifies and approves potential standards for each component of the DHCI, generating a list of possible standards that meet the requirements specified in phase 1. The selected criteria are then used to determine standards for the eHealth CI, which may change depending on the health environment’s resource constraints. The standards determination process involves determining if any international standard can be adopted for each domain, adopting existing global standards, or developing new country-specific standards. The output is standards with developed features such as code, statements, rationales and implementation specifications. The standards are presented to eHealth stakeholders and the public for consultation and validation. The standard development workgroup also develops validation criteria for the standards, focusing on completeness, usability, applicability and perceived usefulness. Phase 2 concludes with consensus building, leading to the approval of the standards. All approved standards are properly documented for publication and widespread sharing with all eHealth stakeholders, either free of charge or at a fee determined by the MoH.

#### Phase 3 of the standards implementation phase

The MoH should collaborate with implementing parties to ensure widespread implementation of mandatory standards. This includes a phased implementation plan, monitoring compliance with agreed standards, and regular review and maintenance of the standards. The key components of this plan include a standard dissemination strategy, a full rollout plan and a compliance monitoring plan. The most critical elements of Phase 3 are actual implementation, monitoring compliance, and keeping up with technological, user needs, and changes in the healthcare environment. The framework presents a monitoring and evaluation (M&E) strategy (see Fig. 2). Monitoring should be a continuous process involving collaborative effort of all implementers and the DH standards’ governing body in the country. Appropiate implementation infrastructure of the DHCI standards should ensure that operational support and resources are well aligned, including staffing needs. Moreover, there should be collaborations at every layer of implementation to ensure right from the first line to national level implementers receive necessary support. Although a detailed discussion of a comprehensive implementation infrastructure is outside the scope of this paper, the study recommends that considering the resource constraints prevalent in LMICs, countries should use existing MoH/DH structures to support DHCI standards implementation and compliance monitoring.

#### Facilitating resources and standardization activities

The framework for eHealth standardization involves key resource mobilization and allocation activities, including the M&E component. The M&E process is continuous and covers all phases of the standardization process. To ensure thoroughness, transparency, openness and accountability, a well-defined M&E team with clear roles and responsibilities, risk assessment criteria, evaluation and review mechanisms, reporting procedures, compliance enforcement mechanisms, and stakeholder sensitization is essential. The framework supports activities such as identifying funds, developing tools and processes, determining required human resources, setting a standardization structure and building human resource capacity to participate in standards development and monitoring compliance. It also includes developing a knowledge base for standards products, supporting policies, laws, regulations, standards implementation guidelines and a human resource book. This ensures due diligence, transparency, openness and accountability in the standardization process.

## RESULTS

### Demographics

**Figure 3:**
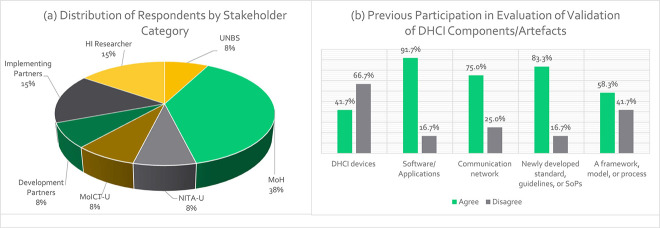
Distribution of stakeholders who validated the framework for standardization of DHCI by (a) category of workplaces and (b) experience in DH evaluation/validation.

Table 1 shows the participant distribution and objective for the framework evaluation. Figure 3 summarizes their distribution and experience in evaluating digital health artifacts. Most respondents (46% MoH officials) were from the HI directorate, 15% were from implementing partners, 15% were researchers and 6% had a bachelor’s or master’s degree in ICT or HI.

Furthermore, Fig. 3(b) shows that 41.7% of the participants in the evaluation of the DHCI framework had previously evaluated/validated DHCI devices; 91.7%, software/applications; 75%, communication network; 83.3%, standards/SoPs; and 58.3%, framework, model or process validation.

### Validation results

The validation process used metrics of *completeness*, *correctness*, *usability*, *perceived usefulness* and *efficacy/satisfaction* to assess the developed DHCI standardization framework. Results from validating the five major constructs of the framework are presented as follows.

### Context setting

**Figure 4:**
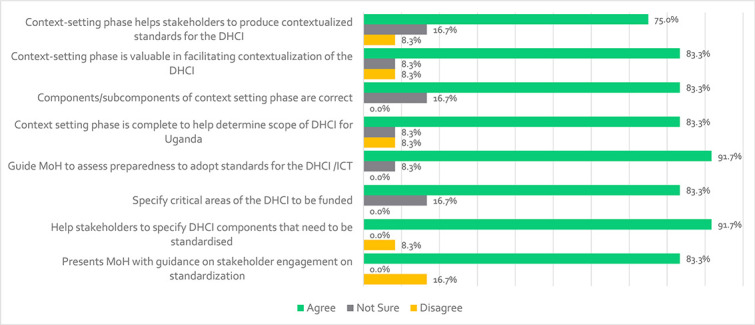
Participants’ views regarding the context-setting phase of the standardization framework

Results in Fig. 4 show that the context-setting phase in Uganda’s DHCI has been validated by most participants, with 75% agreeing it helps stakeholders produce contextual standards. A total of 83.3% believe it supports stakeholder engagement, identifies critical areas for funding, and ensures the components are correct and complete, which is therefore valuable in contextualizing the DHCI. However, a respondent complained that ‘*the framework includes consultation from stakeholders during standards validation, it does not include involvement of key stakeholders (apart from the standards development team) in the early stages of context setting and requirements derivation*’-VR07. Another respondent stated that ‘*All stakeholders should be brought on board*’. The standards development team involves all relevant stakeholders in the standards context-setting phase, including readiness assessment, requirements determination and all subconstruct of the phase. A total of 91.7% agreed that assisting stakeholders in identifying components of the DHCI that need to be standardized and the context-setting phase can help MoH assess preparedness to adopt standards for the DHCI and/or ICT to be used in the healthcare system. Participants also stated that the context-establishing phase should assist in gathering information on existing national, regional and international standards for the DHCI that can be adopted/contextualized since this would ensure that only relevant DH standards are considered in the standardization process (VR01 and VR02).

### Standards contextualization and or development

Next, questions about the standards contextualization/development, public consultation, validation and approval and documentation were asked to assess the three significant subconstruct of the standards contextualization phase. Results show that:

(i) ***Contextualization or development*** 

**Figure 5:**
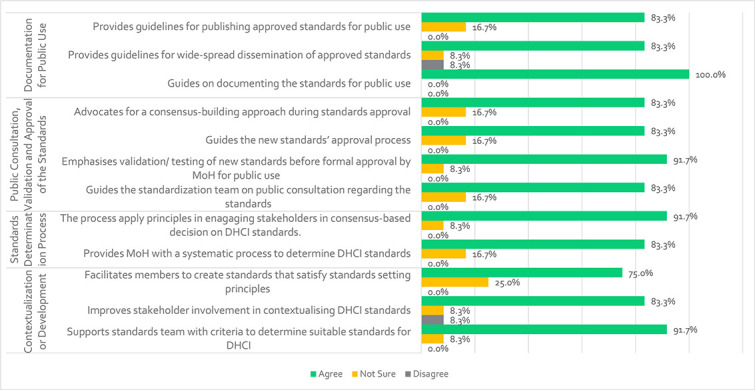
Participants’ views regarding the contextualization activity of the standardization framework

Figure 5 shows that 91.7% of respondents believe that the phase helps the standardization team determine appropriate standards for the DHCI. Furthermore, 83.3% believe that in addition to offering a systematic method for selecting/determining DHCI standards, the phase increases stakeholder engagement in contextualizing/developing DHCI standards.

Finally, 75% of respondents believe that at this stage, participants of the standardization process are encouraged to develop DHCI standards that adhere to the standards-setting criteria of clarity, well-definedness and precision, among others. Slight disagreement with metrics from the standards contextualization phase could be due to doubts expressed by some participants about unanimous agreement on criteria to determine DHCI standards, restrictions on the number of participants in the standardization process, reluctance to apply the process and knowledge of standards setting. However, one participant stated that the composition of the components and the flow of the phase might facilitate the interrelationships of the standards determination group, including the participation of local health system officials.

(ii) ***Standards determination process*** 

The majority of participants (83.3%) agreed that the process was simple enough to guide inexperienced developers in determining suitable standards for the DHCI. The process is suitable for facilitating the selection of DHCI standards for Uganda, supporting the MoH in determining global standards that need local development, and guiding the selection of other DH standards for contextual use in Uganda. The process is complete, providing DH-specific criteria for selecting relevant global standards and guiding decisions on selecting local standards. The process can guide the determination of suitable standards for Uganda’s DHCI, be applied at any health system level, and support different government departments. Furthermore, 91.7% of participants agreed that the process would involve stakeholders in making a consensus-based decision on the DHCI standard. They decided that the framework includes principles to help stakeholders reach a consensus on what standards should be implemented, contextualized or established for Uganda’s DHCI. Furthermore, they agreed that the standards selection process will deliver the expected results, namely contextual criteria for the DHCI that are applicable to Uganda’s health system setting.

(iii) ***Public consultation/validation and approval:*** 

The majority of respondents (83.3%) agreed that the framework directs the standardization team to consult the public when developing standards, provides guidance for the new standards approval process and facilitates consensus-building among DH stakeholders during the standards approval process. Furthermore, 91.3% of respondents believed that the framework guides stakeholders on standard validation/testing before formal approval by the MoH for public usage. No respondents disagreed with any measure.

(iv) ***Documentation for public use*** 

Finally, all stakeholders (100%) agreed that the framework provides guidelines for documenting and publishing approved standards for public use during the standard contextualization phase. A total of 83.3% of respondents agreed that the framework provides guidelines for widespread dissemination and publishing of the standards. However, 8.3% disagree, citing a lack of a specific dissemination mode, possibly due to doubts about the effectiveness of reaching all DH users, including village health teams/community health workers.

### Standards implementation

**Figure 6:**
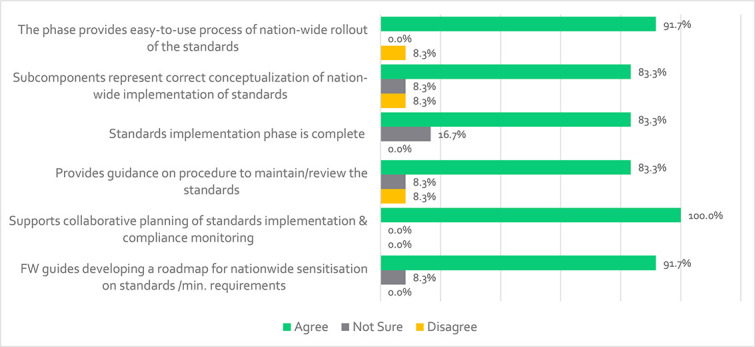
Participants’ views on the standards implementation phase of the standardization framework

Figure 6 shows that all participants (100%) agreed that the phase promotes collaborative planning of coordinated standard implementation and compliance monitoring among entities aiming to participate in the linked health system. A total of 91.7% agreed that the framework provides a road map for educating implementers across the country and an easy-to-use procedure for rolling out the standards nationwide. 83.3% believed that the framework provides a comprehensive set of guidelines for implementation and instructions on maintaining, monitoring and evaluating the standards. Finally, all participants agreed that the framework’s standards implementation phase will be useful in aiding the standardization of the DHCI Uganda’s health system, with 91.7% believing that if properly followed, it will result in establishing a connected health system in Uganda. However, a participant indicated that for the standards to be implemented effectively, the DHCI ‘*standards must be realistic and match what is on the ground*’-VR05, which verifies the study’s conceptualization for standards contextualized to Uganda’s DH resource situation.

### Continuous monitoring and evaluation

**Figure 7:**
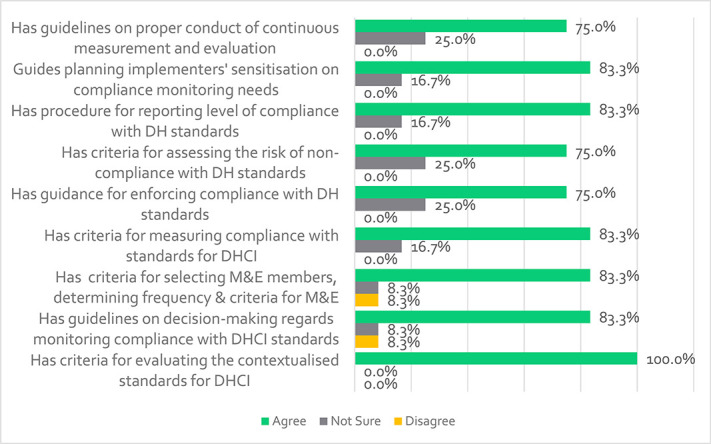
Participants’ views regarding M&E of the DHCI standards

Figure 7 shows that the majority of the participants agree that the framework provides criteria for evaluating contextualized standards for DHCI (100%), guidelines for decision-making regarding compliance monitoring (83.3%), selection criteria for M&E and frequency (83.3%) and criteria for measuring compliance (83.3%). It also offers guidance for enforcing compliance (75%), assessing noncompliance risks (75%), reporting compliance levels (83.3%), planning for implementation sensitization (83.3%), and guidelines for continuous measurement and evaluation of DHCI standards (75%). A participant emphasized that ‘*M&E should be continuous and ensure that there are no loopholes at any stage*’-VR05. Finally, participants agreed that the M&E component would effectively support compliance monitoring (91.7%) and accurately represent the continuous monitoring of DHCI standards (66.7%).

### General issues of the framework for standardization of the DHCI

General issues included assessing the completeness of the overall framework and its potential to guide the standardization of the DHCI in Uganda; most participants agreed on all eight measures.

**Figure 8:**
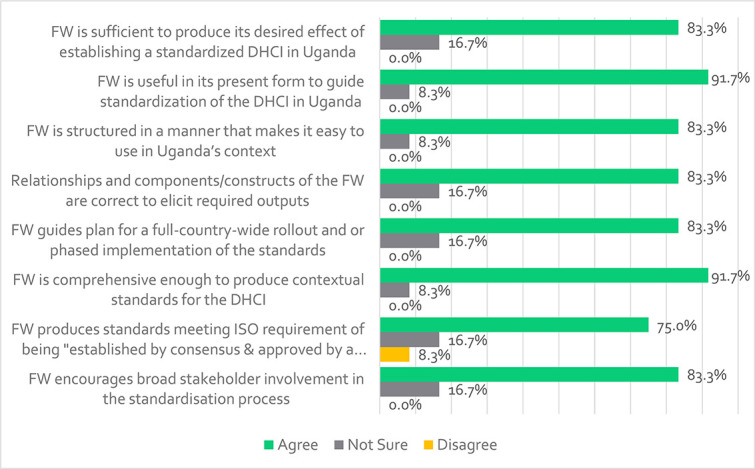
Participants’ views regarding the completeness and applicability of the framework to guide the standardization of the DHCI

Figure 8 shows that most participants agreed that the framework; encourages broader stakeholder engagement in the standardization process (83.3%), helps produce standards that meet ISO requirements of consensus-based (75.0%), is comprehensive in guiding contextualization of standards for DHCI in Uganda (91.7%), guides planning a full-country rollout of standards (83.3%), has correct interrelationship of the components that elicit required outputs (83.3%) and is easy to use (83.3%). In addition, most participants agreed that the framework is useful in its present form to guide the standardization of the DHCI for Uganda (91.3%) and that it is sufficient to produce the desired effect of having a standardized DHCI in Uganda (83.3%). Furthermore, one participant recognized the framework’s comprehensiveness in this statement: ‘*This is a robust framework that covers critical constructs and subconstructs required for standardizing digital health communication infrastructure*’ (VR13). This perspective is a summary of the views of other participants who agreed that the framework was full, accurate and usable for DHCI standardization. This indicates that the framework may be implemented in the Uganda’s DH context. In fact, a respondent (the UNBS Officer) stated, ‘*This framework should be passed on to the national standards body so it can be incorporated into the system of national standards development, leading to the development of a national standard for e-health*’ -VR01. This signifies that participants were satisfied with the framework and agreed that it is usable in its current form. Therefore, the study believes that the framework suits its designated role or purpose (fit-for-purpose). However, one participant pointed out that ‘*the framework highlights review/maintenance of the standard as a key element in phase three; it does not guide on a procedure through which such review/maintenance can be done*’ (VR07). However, it should be noted that the framework’s thorough discourse previously covered a planned standard review procedure, such as when (the review cycle), who (stakeholders to engage in the review process) and how.

## DISCUSSION

In this paper, we present a framework for standardizing the DHCI and validation results of its refined version. The final framework was produced after three cycles of evaluation and improvements. The validation results of version three of the framework showed that;

Respondents were satisfied that all three major phases of context setting, standard development and standard implementation had complete subconstruct to guide standard contextualization and their eventual deployment. To be complete means that no construct is absent; all parts or units must be present and fully developed [33]. Attempts to improve completeness impact the consistency and correctness of the framework. Incorrectness can occur when the descriptions do not accurately reflect the facts or erroneous predictions about future states. However, validation results show that participants were satisfied with all constructs and relationships of the framework, agreeing that they were complete and correct.

Regarding the standards determination process, validation results show that most DH stakeholders in Uganda who responded to the questionnaire agreed that the standards determination process is simple, clear, useful and complete and can be applied in Uganda’s context to produce the desired standards for the DHCI. According to Hevner et al. [23], completeness of activity or function, like the activity-parked standardization process, amounts to functionality. This means that the process provided is simple, clear and useful to provide the required functionality to guide the contextualization/determination of suitable standards for the DHCI.

Respondents agreed that the framework can fast-track the contextualization of standards for the DHCI for Uganda that satisfy International Standards Organization (ISO) principles. The principal advocates for standards to result from a consensus-based agreement among multiple stakeholders and approval by a recognized body [1, 34, 35]. The three-phased framework’s core activities engage and lead to consensus building on standards, dissemination, implementation strategy, M&E and even resource allocation. The framework’s activities extend the SDO’s standard development cycles, including UNBS to implementation, M&E and resource allocation.

To ensure that necessary standards are widely implemented, the MoH should work with affected entities to develop a joint strategy for phased implementation and subsequent compliance monitoring with the standards. This plan’s critical components could include a standards distribution strategy, a thorough deployment plan and a compliance monitoring plan. The most important aspect of Phase 3 is the actual implementation (which may be phased, as directed by the plan), monitoring of compliance with agreed-upon standards, and review/maintenance of the approved standard to keep up with changes in technology, user needs or changes in the healthcare environment.

Respondents raised three key concerns about the standardization framework: stakeholder involvement, standards dissemination and organization of standardization resources. With regard to stakeholder involvement, as advocated for any IS artifact development process following design science research methodology, the framework emphasizes multiple stakeholder engagement throughout the life cycle of the standardization process.

A study of stakeholder involvement in guideline development in clinical practice encourages stakeholder involvement starting from topic selection, panel development, scope determination and prerelease critique [36]. She further claims that involvement may occur through physical participation or representation by stakeholder organizations. According to Balzarova and Castka [37], standards development requires multiple stakeholders to undergo rigorous processes, including eliminating controversial issues: one, a process by which stakeholders articulate potentially unwanted consequences of the new standard. Linking: a process by which stakeholders connect the new standard to existing standards or other documents. Consensus-seeking is a process by which stakeholders encourage and support dialog in standard development. Four, reinforce issues important to the design of a standard: a process by which stakeholders promote their specific position regarding the new standard’s design. Standards improvement: A process by which stakeholders enhance the content of the standard. During the public review of a standard, ‘*anyone is welcome to provide feedback to improve its quality and ensure standards cover all relevant areas and perspectives*’ [38].

In healthcare, dissemination is the targeted distribution of information and intervention materials to a specific public health or clinical practice audience [39]. In medical practice, disseminating guidelines is a prerequisite for its implementation [40]. Therefore, disseminating the contextualized and proven standards should distribute information about the standards among implementing entities to improve existing approaches in healthcare. Just like any guideline in healthcare practice, it is important to communicate the contextualized standards in various contents and formats to increase the likelihood for them to be understood and used by the implementing entities, including distribution of printed materials, publication in professional journals, using electronic media and television and radio broadcasts, among others [39, 40].

Regarding the organization of resources for standardization, the framework already provides guidelines on how the resources can be mobilized and allocated to critical areas. Miller and Kearney [40] identified resources among barriers to compliance with guidelines in the healthcare domain. The dual identified other barriers, including lack of information, culture and limited freedom. Proper allocation of the limited resources available in LMICs could help improve the standardization process from context setting to implementation and compliance monitoring. This process should be well facilitated to improve the framework’s usefulness in guiding the standardization of the DHCI.

Overall, the validation results show that problem owners in Uganda believe that the framework will (i) facilitate broader stakeholder engagement in the standardization process, (ii) help the MoH contextualize DH standards that meet ISO requirements and (iii) guide the MOH in planning the full-country rollout of standards. They agreed that the framework is useful in its present form to guide the standardization of the DHCI for Uganda.

## CONCLUSION

This work followed our earlier field study and literature review that identified several gaps in the DH standardization environment in Uganda as similar LMICs. We developed a standardization framework to aid standards contextualization/development, guide implementation planning and develop a compliance monitoring strategy. This work aimed to contribute to solving an overarching problem of several DH systems lacking in interoperability (semantic, syntactic and technical) and often phased off after the pilot stage by addressing the aspect of technical interoperability through standardizing the DHCI. When assessed, the standardization framework performed well against several evaluation metrics. Overall, respondents agreed that the framework is suitable for use and recommended its immediate adoption to guide standardization efforts in Uganda’s health system. The developed framework for standardizing the DHCI can be used by Uganda (the country of the case study health system) and by any country with similar standardization challenges as those experienced in Uganda. We also believe that the country’s standardization body can adopt the framework to facilitate standardization in domains other than DH. Therefore, as a proof of concept, the researcher will use the framework to contextualize standards for the DHCI for Uganda.

## Supplementary Material

Supplementary_Material_oqae018

## Data Availability

Primary data gathered during framework validation and used to support this paper will be shared on reasonable request with the corresponding author.
